# Dietary Conjugated Linoleic Acid-Enriched Cheeses Influence the Levels of Circulating n-3 Highly Unsaturated Fatty Acids in Humans

**DOI:** 10.3390/ijms19061730

**Published:** 2018-06-11

**Authors:** Elisabetta Murru, Gianfranca Carta, Lina Cordeddu, Maria Paola Melis, Erika Desogus, Hastimansooreh Ansar, Yves Chilliard, Anne Ferlay, Catherine Stanton, Mairéad Coakley, R. Paul Ross, Giovanni Piredda, Margherita Addis, Maria Cristina Mele, Giorgio Cannelli, Sebastiano Banni, Claudia Manca

**Affiliations:** 1 Dipartimento Scienze Biomediche, Università degli Studi di Cagliari, 09042 Monserrato, Italy; m.elisabetta.murru@gmail.com (E.M.); giacarta28@gmail.com (G.C.); lina.cordeddu@ki.se (L.C.); mpmelis@unica.it (M.P.M.); erides82@yahoo.it (E.D.); hastiansar@gmail.com (H.A.); claumanca@hotmail.com (C.M.); 2 Université Clermont Auvergne, INRA, VetAgro Sup, UMR Herbivores, F-63122 Saint-Genès-Champanelle, France; yves.chilliard@inra.fr (Y.C.); anne.ferlay@inra.fr (A.F.); 3 APC Microbiome Ireland, Teagasc Food Research Centre, Moorepark, Fermoy, P61 C996 Co. Cork, Ireland; catherine.stanton@teagasc.ie (C.S.); mairead.coakley@teagasc.ie (M.C.); 4 APC Microbiome Ireland, University College Cork, T12 YT20 Cork, Ireland; p.ross@ucc.ie; 5 Servizio per la Ricerca nei Prodotti di Origine Animale, AGRIS Sardegna, Loc. Bonassai, 07100 Sassari, Italy; gpiredda@tiscalinet.it (G.P.); maddis@agrisricerca.it (M.A.); 6 Fondazione Policlinico Universitario A. Gemelli IRCCS, 00168 Roma, Italy; mariacristina.mele@unicatt.it (M.C.M.); g.cannelli@libero.it (G.C.)

**Keywords:** conjugated linoleic acids (CLA), cheese, ruminant, highly unsaturated fatty acids (HUFA), fatty acids (FA), plasma, clinical trial

## Abstract

n-3 highly unsaturated fatty acids (n-3 HUFA) directly and indirectly regulate lipid metabolism, energy balance and the inflammatory response. We investigated changes to the n-3 HUFA score of healthy adults, induced by different types and amounts of conjugated linoleic acid (CLA)-enriched (ENCH) cheeses consumed for different periods of time, compared to dietary fish oil (FO) pills (500 mg, each containing 100 mg of eicosapentaenoic and docosahexaenoic acids—EPA+DHA) or α-linolenic acid (ALA)-rich linseed oil (4 g, containing 2 g of ALA). A significant increase in the n-3 HUFA score was observed, in a dose-dependent manner, after administration of the FO supplement. In terms of the impact on the n-3 HUFA score, the intake of ENCH cheese (90 g/day) for two or four weeks was equivalent to the administration of one or two FO pills, respectively. Conversely, the linseed oil intake did not significantly impact the n-3 HUFA score. Feeding ENCH cheeses from different sources (bovine, ovine and caprine) for two months improved the n-3 HUFA score by increasing plasma DHA, and the effect was proportional to the CLA content in the cheese. We suggest that the improved n-3 HUFA score resulting from ENCH cheese intake may be attributed to increased peroxisome proliferator-activated receptor alpha (PPAR-α) activity. This study demonstrates that natural ENCH cheese is an alternative nutritional source of n-3 HUFA in humans.

## 1. Introduction

The effects of n-3 fatty acids (n-3 FA) on human health have been extensively studied, and the use of health supplements containing these fatty acids is increasing [1]. In fact, n-3 and n-6 FA are essential lipids that cannot be synthesized de novo and consequently need to be ingested as part of the diet. In humans, the precursor of the n-6 FA, linoleic acid (LA, 18:2n-6), and that of the n-3 FA families, α-linolenic acid (ALA, 18:3n-3), are elongated and desaturated to their more highly unsaturated forms (HUFA), which are n-3 and n-6 FA with ≥20 atoms of carbon and ≥3 double bonds, i.e., principally, arachidonic acid (ARA, 20:4n-6) and docosahexaenoic acid (DHA, 22:6n-3) [2]. There is competitive inhibition between families for enzymatic mechanisms, with, for example, delta-6-desaturase favoring the conversion of n-3 FA over that of n-6 FA [3,4]. However, a high LA intake may shift the balance towards the conversion of n-6 FA and can inhibit the conversion of ALA to eicosapentaenoic acid (EPA, 20:5n-3) or DHA [5].

In humans, this synthesis is a controversial issue, as an ALA-rich diet does not necessarily lead to increased tissue DHA. Healthy subjects receiving 30 mL/day of ALA-rich linseed oil for four weeks demonstrated a 70% increase in DHA in the serum phospholipids [6]. In a long-term study of elderly subjects, the intake of 3 g/day of ALA increased the plasma DHA levels by 20% by 10 months, but not by four months [7]. In contrast, the supplementation of ALA, up to 15 g/day for four weeks, led to increased ALA and EPA levels in plasma triacylglycerols and phospholipids, with very little increase in the DHA levels in plasma, platelets, and white and red blood cells [8,9,10]. Also in other studies, a high dose of ALA for a prolonged period (up to 40 g/day for up to 42 weeks) significantly increased plasma EPA, but not DHA [11,12]. Pawlosky and colleagues studied the conversion of 1 g oral dose of an isotope tracer of ALA, namely, penta-deuterated ethyl ester [13]. They indicated that only 0.2% of plasma ALA was destined for the synthesis of EPA, around 63% of plasma EPA was accessible for the production of docosapentaenoic acid (DPA, 22:5n-3), and 37% of this was available for the synthesis of DHA. In addition, the correlation between DHA and its direct precursor, 24:6n-3, was studied. In cultured skin fibroblasts, the preferred metabolic fate of 24:6n-3-CoA, after its synthesis in the endoplasmic reticulum, is its transfer to peroxisomes, where it is beta-oxidized to DHA-CoA [14].

The n-3 and n-6 HUFA are involved in various physiological processes and influence human health in many different ways [15]. DHA, EPA, and ARA are important components of neuronal membranes [16,17]. DHA is the major HUFA in the adult mammalian brain and retina, and its deficiency can lead to memory loss, learning disabilities, and impaired visual acuity [18]. EPA contributes to cardiovascular health [1,19]. ARA and EPA are precursors of eicosanoids, pro-inflammatory eicosanoids, such as the 2-series prostaglandins, 2-series thromboxanes, 4-series leukotrienes, and hydroxy fatty acids, and anti-inflammatory eicosanoids, such as 3-series prostaglandins, 3-series thromboxanes, and 5-series leukotrienes [3,20,21,22]. A number of studies have provided strong evidence that the resolution of inflammation is a biosynthetically active process, regulated by biochemical mediators and receptor-dependent signaling pathways, driven by specialized pro-resolving mediators [23]. These specialized pro-resolving mediators lead to the resolution of inflammatory processes and restore homeostasis. [24].

With current dietary practices, our dietary n-6/n-3 HUFA ratio is elevated for the overall population, with the optimal ratio being 4:1 [25,26]. However, this imbalance can be corrected by dietary supplementation with n-3 HUFA or by eating fish rich in EPA and DHA, such as salmon, herring, mackerel, and tuna [27]. Recommendations are in the range of 0.5–0.8 g/day of n-3 HUFA, or the consumption of at least two servings of fish per week [28]. A physiological marker of n-3 FA in tissues is the n-3 HUFA score obtained as the ratio between the n-3 HUFA (FA ≥ 20 carbons and ≥3 double bonds) and the total FA; HUFA are mainly incorporated into plasma lipids (phospholipids) and are potential precursors of the biologically active eicosanoids and docosahexaenoids [29].

Functional foods, nutraceuticals or food products with enhanced n-3 HUFA levels are becoming more popular, as a result of innovative development strategies. Another option to enrich for n-3 HUFA in humans may be the incorporation of flaxseed products in livestock feed, resulting in the accumulation of HUFA in tissues and other animal products (milk, cheeses or meat). Pasture and/or lipid supplementation of ruminants’ diet was used to increase by up to four-fold milk fat ALA, a precursor of n-3 HUFA, the c9t11 isomer of conjugated linoleic acid (CLA, 18:2*c*9*t*11), and vaccenic acid (VA, 18:1*t*11) and to decrease by 23% the saturated fatty acids (SFA) [30,31,32,33].

CLA, an unusual fatty acid characterized by conjugated double bonds [34], is synthetized within the rumen as a result of the activity of anaerobic bacteria [35,36], and the majority of the c9t11 isomer of CLA is endogenously synthesized in the mammary gland of ruminants [33] and also in humans from VA [37,38].

The c9t11 isomer of CLA is metabolized in a similar manner to LA and ALA, competes with them for the same enzymatic systems, and plays a role in lipid metabolism [39]. In a previous study on hypercholesterolemic subjects, we demonstrated that the intake of 90 g/day of a functional food (a CLA-enriched (ENCH) cheese), was an efficient approach to enhance the n-3 HUFA score [40]. Interestingly, improved lipid metabolism was achieved with a 90 g/day and not with a 45 g/day intake of ENCH cheese, suggesting a dose-dependent effect, at least after three weeks of dietary intervention [40].

In this study, we investigated the plasma fatty acid profiles of healthy human volunteers and evaluated the effect of naturally CLA-enriched cheese on n-3 FA metabolism to optimize the n-3 HUFA scores. We compared the modifications in the plasma fatty acid profiles following different daily intakes of ENCH cheese (90 g/day) for two and four weeks, or 50 g/day of cheese for two months. In a longer treatment, we compared the effects of different types of ENCH cheeses, produced from the milk of three different ruminant species. The effects of a fish oil (FO) supplement or of ALA-containing linseed oil were compared to those of ENCH cheese.

## 2. Results

The pilot study consisted of 15 healthy human subjects fed 90 g per day of an ENCH cheese (sheep-1-ENCH) for an initial two-week period; plasma fatty acids were then measured (Table 1), and the feeding continued for a further two weeks. The plasma concentrations of palmitic acid (PA, 16:0) were markedly reduced in the subjects over the four weeks of the dietary intervention compared to time zero (baseline). A daily intake of 90 g/day of sheep-1-ENCH cheese resulted in a significant increase in EPA and DHA plasma levels corresponding to 80% and 20%, respectively, by week 4 (Table 1). In addition, there was a greater incorporation of CLA as a result of sheep-1-ENCH cheese intake, leading to an increase of 220% after two weeks and of 240% after four weeks compared to the baseline (Table 1).

**Figure 1:**
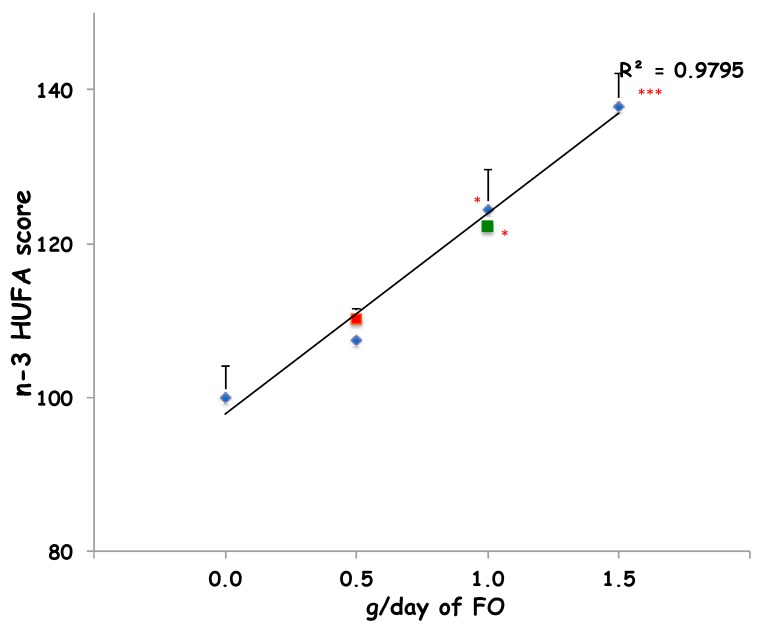
Plasma n-3 highly unsaturated fatty acids (HUFA) scores of subjects fed with 0.0, 0.5, 1.0, and 1.5 g/day of fish oil (FO) (◆ blue diamond) or 90 g/day of sheep-1-ENCH cheese for two weeks (■ red squares) and four weeks (■ green squares). The data are expressed as mean ± SEM per group; * *p* < 0.05; *** *p* < 0.001 versus baseline (time 0).

Furthermore, the intake of 90 g/day of sheep-1-ENCH for two and four weeks improved the n-3 HUFA score (from 11% to 28%, respectively) when compared to the baseline (Figure 1). In order to compare the impact on the plasma n-3 HUFA score of 90 g/day of sheep-1-ENCH cheese with that of increasing FO supplementation (0.5, 1 and 1.5 g per day), the subjects were treated for two weeks with one of the indicated FO doses, which resulted in a linear plasma incorporation of n-3 HUFA in a dose-dependent manner (Figure 1). The n-3 HUFA scores achieved with a daily intake of 90 g of sheep-1-ENCH cheese for two and four weeks corresponded to those obtained with a daily FO ingestion of 0.5 g FO (one supplement) and 1.0 g FO (two supplements) per day, respectively (Figure 1).

**Figure 2:**
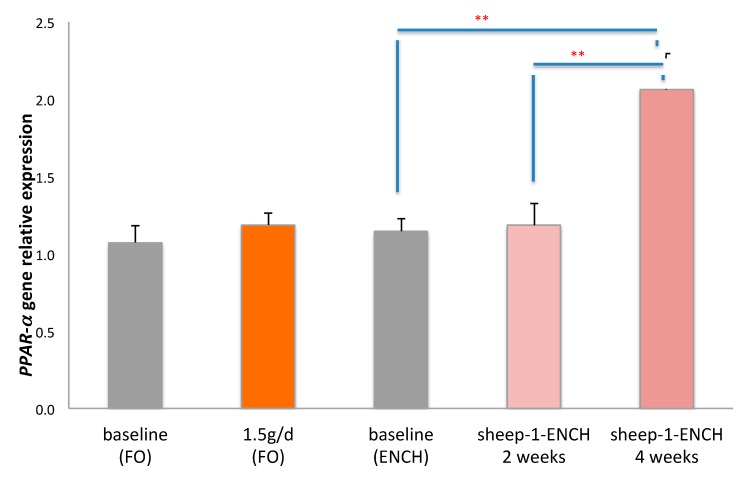
Relative *PPAR-*α gene expression in the buffy coat of subjects consuming 1.5 g/day of FO and 90 g/day of sheep-1-ENCH cheese for two and four weeks. The data are expressed as mean ± SEM, with 15 individuals per group; ** *p* < 0.01; versus baseline (time 0).

Since n-3 HUFA, particularly DHA and CLA, are PPAR-α ligands [41], we also evaluated the relative expression levels of the *PPAR-*α gene. *PPAR-*α gene expression analysis indicated a significant two-fold increase following dietary intervention with 90 g/day of sheep-1-ENCH cheese for four weeks (*p* < 0.01) when compared to the baseline or to the intake for two weeks of sheep-1-ENCH cheese (*p* < 0.01). However, there were no significant differences following the intake of the FO supplement at 1.5 g/day (Figure 2).

We evaluated whether the results obtained in the pilot study with a sheep-1-ENCH cheese were reproducible during a longer period (two months) and with different ruminant ENCH cheeses of bovine, ovine and caprine origin. In this experiment, 50 g per day of cheese was ingested for two months, as the amount consumed in the pilot study (90 g/day) may be considered high for the longer feeding period. This lower intake was calculated on the basis of the regular intake of cheese in the Italian diet [42].

Table 2 outlines the plasma FA levels in subjects ingesting ENCH cheeses (50 g per day) from different sources.

The plasma levels of ALA and CLA increased in all subjects after ENCH cheese intake when compared to the control values. There was a significant increase in the incorporation of CLA (>280%) into the plasma of subjects ingesting goat-ENCH cheese compared to subjects consuming the control (CRTL) cheese. A similar result was observed for the cow-ENCH cheese (>140% increase in CLA) when compared to the CRTL values.

These high plasma CLA levels detected in the subjects consuming goat-ENCH cheese reflected the 1.5-fold greater CLA concentration in goat-ENCH cheese with respect to other ENCH cheeses (see Table 2). No significant variations were found in the plasma levels of ALA and EPA when the different ENCH cheeses were consumed; only the DHA levels increased by ~24–37% following the intake of all types of ENCH cheeses (see Table 2).

**Figure 3:**
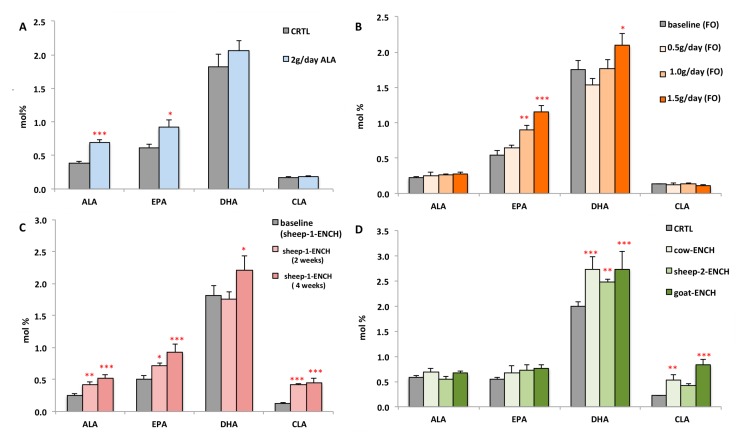
Levels of the main n-3 FA (ALA, EPA, and DHA) and CLA measured in the plasma of subjects consuming different diets: (**A**) CRTL diet (4 g/day olive oil) or 4 g/day linseed oil (~2 g of ALA) for two months, *n* = 12/group; (**B**) 0.5 g/day, 1.0 g/day, or 1.5 g/day of FO for two weeks, *n* = 15/group; (**C**) 90 g/day of sheep-1-ENCH cheese for two and four weeks, *n* = 15/group; (**D**) 50 g/day of different ENCH or CRTL cheeses for two months, *n* = 36/group. The data are expressed as mol % and represent mean ± SEM per group; * *p* < 0.05; ** *p* < 0.01; *** *p* < 0.001 versus baseline (time 0) or control group.

We also evaluated whether a substantial intake (2 g) of the n-3 HUFA precursor (ALA) significantly modified the plasma DHA levels and, hence, impacted the n-3 HUFA scores. Subjects administered for two months 4 g/day of linseed oil, containing 2 g of ALA, were compared with subjects administered 4 g/day of olive oil (control group). We observed a significant rise in ALA and EPA (78% and 50%, respectively), but not of DHA (13% increase, Figure 3A) in the linseed oil group. Figure 3 compares the plasma levels of ALA, EPA, DHA, and CLA following the different treatments of this study. It was observed that high circulating CLA levels induced DHA biosynthesis more strongly than the DHA precursor ALA.

**Figure 4:**
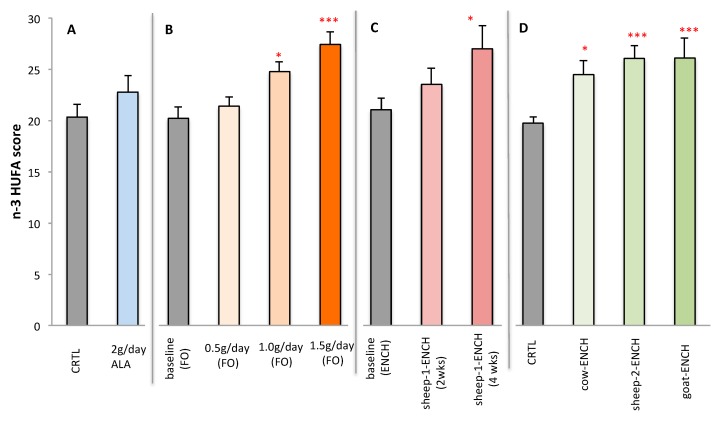
Plasma n-3 HUFA scores of subjects administered different treatments: (**A**) CRTL control (4 g/day olive oil) and 4 g/day linseed oil (~2 g of ALA), *n* = 12/group; (**B**) 0.5 g/day, 1.0 g/day and 1.5 g/day of FO for 2wo weeks, *n* = 15/group; (**C**) 90 g/day of sheep-1-ENCH cheese for two and four weeks, *n* = 15/group; (**D**) 50 g/day of different ENCH or CRTL cheeses for two months, *n* = 36/group. The data are expressed as mean ± SEM per group; * *p* < 0.05; *** *p* < 0.001 versus baseline (time 0) or control group.

Figure 4 represents the plasma n-3 HUFA scores of subjects on the different treatments. Significant increases were observed with the intake of ENCH cheese for longer periods or with the higher concentrations of FO.

## 3. Discussion

Recently, attention has been focused on dietary regimens aimed at meeting the optimal nutritional lipid requirements to exert positive effects on health and to prevent the onset of chronic diseases such as cardiovascular diseases, diabetes, metabolic syndrome and cancer [43]. The beneficial effects of n-3 FA have been widely described within an optimal balance of all dietary FA [15]. It is likely that the putative beneficial effects exerted by n-3 FA are dictated by the general deficiency of EPA and DHA intake in Western European countries [44]. In particular, it has been proposed to reduce the n-6/n-3 HUFA ratio to the desired optimal value of 4:1 by regulating the dietary intake of the corresponding HUFA.

The aim of this study was to evaluate whether a functional food, such as a CLA-enriched cheese, was able to address the imbalance between n-6 and n-3 fatty acid families in healthy subjects on a regular diet. We previously observed that feeding sheep-1-ENCH cheese increased the plasma n-3 HUFA score, one of the more reliable indicators of n-3 FA status in humans [29,45] and in hypercholesterolemic subjects [40]. In that study, we also found a significant reduction of LDL-cholesterol and of the endocannabinoid anandamide [40]. In the present study, cheese intake was not associated with changes in body weight, plasma lipid content or endocannabinoids (data not shown). These data may suggest that the effects of ENCH cheese on LDL-cholesterol and endocannabinoids levels are significant only in altered physiopathological conditions, such as those of hypercholesterolemic patients, and are not due to a direct influence on cholesterol metabolism or endocannabinoid biosynthesis.

We found that the intake of ENCH cheeses resulted in a significant increase in plasma DHA in humans ingesting either 90 g per day of sheep-1-ENCH cheese for four weeks or 50 g per day of an ENCH cheese from three ruminant species for two months. These data may suggest that not only the amount but also the duration of the ENCH cheese intake are crucial to induce DHA biosynthesis. We previously found that CLA intake in Zucker rats for 14 weeks increased the n-3 HUFA score in the liver [46]. Several studies have examined the ability of CLA to increase the peroxisomal or mitochondrial activity of fatty acid oxidation, and CLA is a potent ligand of the peroxisome proliferator-activated receptor alpha (PPAR-α) that regulates expression of the genes involved in peroxisomal beta-oxidation [47]. The importance of the peroxisomal beta-oxidation process was previously described in relation to DHA synthesis [14]. We may hypothesize that CLA could induce higher DHA formation via PPAR-α activation, but further studies are needed to demonstrate a correlation between CLA and improved DHA synthesis. Indeed, we found that sheep-1-ENCH cheese (90 g/day) significantly increased *PPAR-α* gene expression. In addition, our data suggest that this enhanced gene expression is not just due to DHA, since FO, in this study, did not demonstrate any significant effect on *PPAR-α* gene expression. Our data were supported by other studies in experimental animals that detailed the ability of CLA to activate PPAR-α and thus to induce key enzymes of peroxisomal beta-oxidation [47,48].

Attar-Bashi and collaborators showed that CLA did not efficiently increase DHA levels from ALA [49]. Experimental animals models and human studies have shown that high dietary ALA partially inhibits delta-6 desaturase and thus prevents the desaturation of 24:5n-3 to 24:6n-3 in the DHA biosynthesis pathway [50]. ALA can be endogenously converted into its metabolites at a very low efficiency, 5% to EPA and around 0.5% to DHA [51,52]. Our previous data demonstrated a reduction of DHA in the brain of rats fed an ALA-enriched diet and an increase of the other precursor (22:5n-3) [53]. From our data, it appears that for CLA to exert its effect on circulating DHA levels, a relative but not excessive intake of ALA is necessary. We propose that a ratio of dietary CLA/ALA of ~3:1 could be determinant to enhance DHA production from ALA. The role of CLA at certain concentrations, with respect to ALA, was further demonstrated by the prolonged intake of ALA-rich linseed oil, which only slightly induced a non-significant increase in the n-3 HUFA score. Moreover, the intake of the sheep-1-ENCH cheese resulted in plasma n-3 HUFA scores comparable to the intake of fish oil. This effect further confirms the notion that food composition may not reflect food nutritional value. Often, the nutritional guidelines for dietary fat intake rely on food fatty acid composition and, for example, a certain concentration of ALA is sufficient to claim that a food is a source of n-3. Our data clearly demonstrate that this is not the case and that an increase in circulating DHA may be efficiently achieved with different strategies, not only by supplying dietary n-3 FA. Interestingly, we found a significant decrease of PA, the major SFA in plasma, with the intake of ENCH cheeses. This might be surprising since cheese is sometimes negatively perceived because of its elevated SFA levels [54]. It is known that dietary PA does not influence PA tissue levels in humans, as it is regulated by the balance between its main sources, i.e., diet and biosynthesis, through de novo lipogenesis (DNL) [55], which is a very well conserved mechanism converting carbohydrates to lipids [56]. We may hypothesize that ENCH cheeses may inhibit DNL probably by activating PPAR-α [57], thereby reducing the circulating PA levels.

The activation of PPAR-α with the inhibition of DNL and the increase of the n-3 HUFA score may, at least in part, explain the anti-neuroinflammatory effects exerted by CLA in X-linked adrenoleucodystrophy, a genetic disorder characterized by an impaired peroxisomal beta-oxidation [58,59], confirming that PPAR-α activation can also play an important role in the prevention of neurodegenerative pathologies [60,61]. The development of innovative systems in livestock breeding aimed at increasing milk CLA content would produce dairy products rich in CLA, which, if included into a balanced diet, may contribute to the prevention of some of the major chronic pathologies [62]. The supposed health benefits of CLA were discovered nearly three decades ago [63]. Such positive health effects were related to cancer suppression, body fat reduction, delay of the onset of type II diabetes and of the development of atherosclerosis, improved bone mineralization, and immune system modulation [64,65,66]. Interestingly, these beneficial effects are shared with n-3 HUFA.

In conclusion, our studies indicate that ENCH cheeses may contribute, with other nutritional metabolic and genetic factors, to the modulation of fatty acid metabolism. Because of the limited habitual consumption of seafood in most Western countries, if our data were confirmed in larger multicenter studies, ENCH cheeses may be included in the guideline recommendations to ensure a sufficient supply of n-3 HUFA. Future studies will be devoted to the evaluation of the possible synergistic effects of CLA-enriched products and other dietary components.

## 4. Materials and Methods

### 4.1. Subjects

All protocols involving human subjects were performed according to Good Clinical Practice with pre-approval from the appropriate Ethics Committee. This study was carried out at the Catholic University of the Sacred Heart, Rome (Italy). A total of 36 apparently healthy individuals, 16 males and 20 females, aged 30–45 years, with Body Mass Index (BMI) 20–26, and blood glucose and lipids parameters shown in Table 3, were included in the study. Among the 36 participants, 15 were randomly assigned to Studies 1 and 2, 24 to Study 3, and all 36 were recruited for Study 4.

The use of alcohol, tobacco and medicines that might influence the absorption or metabolism of lipids, was not permitted during the studies, and the subjects were advised to maintain their customary living habits, in particular in relation to diet.

### 4.2. Blood Sampling

Following overnight fasting, venous blood samples were taken from the antecubital vein into anticoagulant-coated vacutainers (K3 EDTA) and centrifuged at 2000× *g* for 15 min at room temperature. The plasma was separated from the red blood cells and stored at −80 °C until quantification of total lipids and fatty acids profiling. The buffy coat was also separated, stabilized in RNA Later (Ambion, Life Technologies, Carlsbad, CA, USA) and stored at −20 °C for RNA extraction.

### 4.3. Study Design

**Study 1: Feeding CLA-enriched cheese (ENCH)**

For two and four weeks, the diet of 15 healthy subjects was supplemented with 90 g/day of a sheep cheese, enriched in ALA and CLA (sheep-1-ENCH), kindly provided by Argiolas Formaggi s.r.l (Dolianova, Italy). The daily intake (90 g/day) of sheep-1-ENCH cheese was as published in our previous paper [40]. CLA-enriched milk was obtained from dairy ewes on pasture when available or by supplementing the diet with extruded linseeds. [40].

The subjects visited the clinic on three occasions: (a) for the base examination (time 0); (b) at the end of the intake of 90 g/day of sheep-1-ENCH cheese for two weeks; (c) following the intake of 90 g/day of sheep-1-ENCH cheese for four weeks.

**Study 2: Feeding fish oil (FO) supplements with n-3 HUFA**

Following a three-weeks washout (WO) period, the diet of the subjects from Study 1 were supplemented with stepwise increasing doses of FO in pill form (containing 14.6% EPA, 7.8% DHA, 25.9% oleic acid (OA, 18:1n-9), 10.2% LA and 0.9% ALA, with respect to total FA); kindly supplied by Aker Biomarine (Oslo, Norway).

The subjects consumed: (a) 0.5 g/day FO supplement for two weeks; (b) 1 g/day FO supplement for two more weeks and (c) 1.5 g/day FO supplement for a further two-week period, corresponding to one, two and three pills per day, respectively.

**Study 3: Effect of feeding α-linolenic acid (ALA), a precursor of the n-3 HUFA family**

Twenty-four healthy subjects were randomly divided into two groups. The diet of one group was supplemented for two months with 4 g of linseed oil (rich in ALA) corresponding to an intake of 2 g/day of ALA, and the diet of the other group was supplemented with 4 g/day of olive oil (control).

**Study 4: Feeding ENCH cheese from different ruminant sources**

The diets of 36 healthy volunteers were supplemented for two months with different types of ENCH cheese or with a control cheese, produced from the milk of three ruminant species (bovine, ovine and caprine).

The ENCH cheeses studied were produced from:

1. Cow’s milk (cow-ENCH); supplied by Teagasc, Moorepark, Fermoy, Co. Cork, Ireland. CLA was increased in the milk by supplementation with linseed and sunflower oils (unpublished data).
2. Sheep’s milk (sheep-2-ENCH); Pecorino Sardo PDO cheese supplied by AGRIS, Bonassai, Olmedo, Italy, which was enriched in CLA by using a sheep feeding regimen characterized by extensive grazing of grass–legume pastures and low levels of supplements [67].
3. Goat’s milk (goat-ENCH); supplied by INRA, Clermont-Ferrand-Theix, France. The CLA-enriched goat spread-type cheese was manufactured by Actalia (Surgères, France). The milk was enriched in CLA as a result of an alfalfa hay-based diet supplemented with sunflower oil (130 g/day) and Vitamin E (50 g/day) [68].

The FA profiles of the control and ENCH cheeses are detailed in Table 4. The values of the control cheese are the mean of three control cheeses.

The content of CLA was higher in the ENCH cheeses than in the control cheeses and, in particular, it was three times higher in the cow and sheep cheeses, and five times higher in the goat cheese. The content of VA was three times higher in the ENCH cheeses. In contrast, the content of ALA was extremely variable, and the ALA content of the goat cheese was similar to that of the control cheese.

The subjects were randomly assigned to one of the following two groups and a double-blind procedure was applied. Each subject received 50 g/day of edible control (CRTL) or ENCH cheese, for two months. Following a two-month WO period, the cheeses were alternated, such that the control group ingested the ENCH product and *vice versa*, for a further two months. Following a further WO period, the study was repeated with the same subjects, according to the experimental design outlined above, using different cheeses (always ENCH and CRTL), provided by another producer. With this treatment, each subject consumed each type of control and ENCH cheeses.

Blood samples were collected from each participant before and after the cheese intake periods and after the WO periods, and total lipids and the fatty acid profiles were analyzed.

### 4.4. Lipid Analyses

Plasma total lipids were extracted with a chloroform/methanol 2:1, (*v*/*v*) solution [69] and were quantified as outlined by Chiang et al. [70]. The total lipids were extracted by the method of Folch. Briefly, samples of human plasma were dissolved in a 2:1 chloroform/methanol solution containing deuterated endocannabinoids and congeners for endocannabinoids quantification: 200 ng of *N*-arachidonoylethanolamine or anandamide ([^2^H]_8_AEA), 300 ng of 2-arachidonoyl–glycerol ([^2^H]_5_2AG), 200 ng of *N*-oleoylethanolamine ([^2^H]_2_OEA) and 100 ng of *N*-palmitoylethanolamine (^2^H]_4_PEA) [71].

An aliquot of the plasma lipid extract was used for HPLC separation to determine the total free fatty acids as chloroform aliquots were mildly saponified [72] and to be analyzed later for fatty acid profile determination by HPLC with an Agilent 1100 HPLC system (Agilent, Palo Alto, CA, USA) equipped with a diode array detector (DAD), as previously reported [73]. SFA are transparent to ultraviolet (UV) light; thus, after methylation, they were measured as fatty acid methyl esters (FAME) by a gas chromatograph (GC) (Agilent, Model 6890, Palo Alto, CA, USA) equipped with a flame ionization detector (FID), an autosampler (Agilent, Model 7673), and a 100 m HP-88 fused capillary column, as previously described by Batetta et al. (2009) [74].

The quantification of the endocannabinoids AEA and 2-AG and of the *N*-acylethanolamide (NAE) compounds OEA and PEA, was carried out by liquid chromatography–atmospheric pressure chemical ionization–mass spectrometry (LC–APCI–MS), using selected ion monitoring (SIM) at M+1 values for the compounds and their deuterated homologs. The internal deuterated standards for AEA, 2-AG, OEA, and PEA quantification by isotope dilution ([^2^H]_8_AEA, [^2^H]_5_2AG, [^2^H]_2_OEA, [^2^H]_4_PEA) were purchased from Cayman Chemicals (Ann Arbor, MI, USA). A C-18 Zorbax Eclipse Plus column (5 μm particle size, 50 mm, 4.6 mm; Agilent, Palo Alto, CA, USA) was used with a mobile phase of CH_3_OH/H_2_O/CH_3_COOH (80/20/0.3, *v*/*v*/*v*) at a flow rate of 0.5 mL/min [71].

The n-3 HUFA score was obtained by calculating the sum of n-3 FA with 20 or more carbon atoms and three or more double bonds, divided by the sum of total fatty acids with 20 or more carbon atoms and more than two double bonds; (EPA + DHA + DPAn3)/(EPA + DHA + DPAn-3 + 20:3n6 + ARA + 22:4n6 + DPAn-6 + 20:3n-9) × 100 [29].

### 4.5. Quantitative Analysis of PPAR-α mRNA

RNA was extracted from the buffy coat samples with the Ribopure Blood Kit (Ambion) following the manufacturer’s instruction and eluted in 100 μL of preheated elution solution. A subsequent DNase I digestion removed contaminating genomic DNA from the eluted RNA. The concentration and purity of RNA was determined by measuring the absorbance of the RNA in a spectrophotometer (Cary 60 UV-Vis Agilent Technologies) at 260 and 280 nm.

In total, 900 ng from each extracted RNA was reverse transcribed with an iScript cDNA synthesis kit (Bio-Rad, Oxford, UK) in a reaction volume of 20 μL, according to the manufacturer’s protocol. The cycling conditions were: 25 °C for 5 min, 42 °C for 30 min, and 85 °C for 5 min. Complementary DNA was used to evaluate the expression of the *PPAR-α* gene. For each sample, quantitative real-time PCR was performed using a sense and an antisense specific primer pair (Bio-Rad, Oxford, UK) for the gene of interest (qHsaCID0011001_intron spanning, Bio-Rad, Oxford, UK) and the SYBR Green method (Bio-Rad, Oxford, UK). The amplification reaction was performed in the MiniOpticon real-time PCR system (Bio-Rad, Oxford, UK). Analysis of the melting curves was also performed. The samples were analyzed in triplicate and, in addition to the target gene, β-actin was used as a reference gene (qHsaCED0003749_exonic, Bio-Rad, Oxford, UK). The target mRNA expression levels were normalized to β-actin expression considering the primer pair efficiencies (93% and 102% for PPAR-*α* and β-actin primer pairs, respectively) and represented as a relative value according to the 2^−ΔΔ*C*t^ method. Each PCR run included a no-template control.

## 5. Statistical Analyses

Data are expressed as the mean ± standard error of the mean (SEM), as specified in the individual tables and figures. The differences between two groups were assessed using an unpaired, two-tailed Student’s *t*-test. Data sets involving more than two groups were assessed by one-way ANOVA followed by a Tukey’s post-hoc test. The differences were considered statistically significant for *p* < 0.05, and different superscript letters indicate significant differences between groups.

The data were analysed using Software GraphPad Prism 6.01 (La Jolla, CA, USA).

## Figures and Tables

**Table 1:** Main fatty acids (FA) in the plasma of subjects consuming 90 g/day of a conjugated linoleic acid (CLA)-enriched (ENCH) sheep cheese (sheep-1-ENCH). PA, palmitic acid; OA, oleic acid; ALA, α-linolenic acid; EPA, eicosapentaenoic acid; DHA, docosahexaenoic acid; LA linoleic acid, ARA, arachidonic acid. The data are expressed as mol % and represent the mean ± SEM for 15 individuals per group; * *p* < 0.05; ** *p* < 0.01; *** *p* < 0.001 versus baseline (time 0).

FA mol % in Plasma	Baseline (Time 0)		Sheep-1-ENCH (2 Weeks)		Sheep-1-ENCH (4 Weeks)	
	Mean	SEM	Mean	SEM	Mean	SEM
14:0	1.25	0.24	1.16	0.16	1.36	0.26
16:0 (PA)	30.28	0.54	29.52	0.34	24.72	0.63 ***
17:0	7.27	0.22	7.49	0.12	6.02	0.21 ***
18:0	1.03	0.06	1.16	0.06	1.03	0.08
16:1	2.54	0.15	2.53	0.22	2.32	0.30
18:1n-9 (OA)	18.20	0.60	18.62	1.53	19.95	1.03
18:3n-3 (ALA)	0.25	0.03	0.42	0.04 **	0.52	0.05 ***
18:4n-3	0.03	0.01	0.03	0.00	0.03	0.00
20:5n-3 (EPA)	0.51	0.05	0.71	0.05 *	0.93	0.13 ***
22:6n-3 (DHA)	1.81	0.15	1.75	0.11	2.51	0.15 *
18:2n-6 (LA)	27.05	0.85	29.68	1.59	31.63	0.62
18:3n-6	0.54	0.05	0.49	0.03	0.49	0.04
20:3n-6	1.52	0.12	1.53	0.19	1.64	0.13
20:4n-6 (ARA)	6.79	0.37	6.46	0.43	7.11	0.38
22:4n-6	0.14	0.02	0.11	0.01	0.11	0.00
18:2c9t11 (CLA)	0.13	0.10	0.42	0.23 ***	0.45	0.07 ***

**Table 2:** Main FA in the plasma of subjects consuming 50 g/day of different types of ENCH cheese for two months. The cheeses were produced from the milk of cows (cow-ENCH), sheep (sheep-2-ENCH) and goats (goat-ENCH). PA, palmitic acid; OA, oleic acid; ALA, α-linolenic acid; EPA, eicosapentaenoic acid; DHA, docosahexaenoic acid; LA linoleic acid, ARA, arachidonic acid; VA, vaccenic acid and CLA, conjugated linoleic acid. The data are expressed as mol % and represent mean ± SEM of 36 individuals per group; * *p* < 0.05; ** *p* < 0.01; *** *p* < 0.001 versus control (CRTL) group.

FA mol % in Plasma	CRTL		Cow-ENCH		Sheep-2-ENCH		Goat-ENCH	
	Mean	SEM	Mean	SEM	Mean	SEM	Mean	SEM
14:0	0.64	0.02	1.31	0.12 ***	0.66	0.06	0.58	0.07
15:0	0.22	0.00	0.42	0.03 ***	0.17	0.01 *	0.22	0.02
16:0 (PA)	11.53	0.25	11.26	2.27	11.13	0.30	11.93	0.85
17:0	0.50	0.02	0.38	0.04	0.34	0.02 **	0.50	0.05
18:0	9.03	0.29	6.90	0.42	6.95	0.21 *	7.89	0.78
14:1	0.09	0.01	0.07	0.01	0.05	0.00	0.07	0.01
16:1	1.82	0.12	2.07	0.24	1.77	0.06	1.57	0.11
18:1n-9 (OA)	26.31	1.02	29.96	1.90	27.48	1.30	26.70	1.19
18:3n-3 (ALA)	0.58	0.03	0.69	0.04	0.54	0.05	0.67	0.08
18:4n-3	0.02	0.00	0.02	0.01	0.02	0.00	0.02	0.00
20:5n-3 (EPA)	0.54	0.03	0.67	0.07	0.73	0.11	0.76	0.14
22:6n-3 (DHA)	1.99	0.09	2.73	0.33 ***	2.47	0.08 **	2.74	0.28 ***
18:2n-6 (LA)	35.58	0.76	40.73	2.47 *	38.67	1.56	34.32	1.24
18:3n-6	0.41	0.03	0.50	0.06	0.39	0.03	0.37	0.05
20:3n-6	1.89	0.08	2.32	0.11 *	1.58	0.09	1.96	0.16
20:4n-6 (ARA)	8.06	0.35	7.79	0.85	7.20	0.31	7.52	0.33
22:4n-6	0.27	0.01	0.31	0.01	0.23	0.02	0.27	0.03
*18:1*t*11* (VA)	0.36	0.02	0.69	0.07 **	0.57	0.06 *	1.03	0.15 ***
18:2c9t11 (CLA)	0.22	0.01	0.53	0.11 **	0.41	0.04	0.85	0.11 ***

**Table 3:** Blood lipid parameters of the study populations: BMI = Body Mass Index; HDL-C = High-density lipoprotein cholesterol; LDL-C = Low-density lipoprotein cholesterol.

Blood Parameters	Mean	SEM
Age (years)	30–40	
BMI (kg/m^2^)	20–26	
Glycemia (mg/dL)	85.5	1.5
Triglyceride (mg/dL)	80.3	6.3
Total cholesterol (C) (mg/dL)	190.5	6.7
HDL-C (mg/dL)	60.1	1.9
LDL-C (mg/dL)	113.8	6.1
Total C/HDL-C ratio	3.3	0.1

**Table 4:** FA profiles (g FA/50 g cheese) of the control and of the cow-, sheep-2-, and goat-ENCH cheeses.

g FA/50 g of Cheese	Mean of CRTL Cheeses	Cow-ENCH	Sheep-2-ENCH	Goat-ENCH
short chain (c4–c10)	2.30	1.55	2.06	2.15
12:0	0.63	0.38	0.50	0.43
14:0	1.33	0.79	1.06	1.11
15:0	0.14	0.14	0.13	0.13
16:0 (PA)	2.98	2.30	2.16	2.27
17:0	0.09	0.05	0.08	0.07
18:0	1.02	1.35	1.20	1.08
14:1	0.05	0.04	0.02	0.01
16:1	0.10	0.11	0.07	0.05
18:1n-9 (OA)	1.85	2.59	1.99	1.98
18:2n-6 (LA)	0.24	0.23	0.20	0.41
18:3n-3 (ALA)	0.09	0.15	0.13	0.04
18:1t11 (VA)	0.19	0.86	0.61	1.06
18:2c9t11 (CLA)	0.07	0.30	0.31	0.48
